# Supplementary material for: Development of a Smoke-Free Home Intervention for Families of Babies Admitted to Neonatal Intensive Care
Source: Int J Environ Res Public Health. 2022 Mar 19;19(6):3670. doi: 10.3390/ijerph19063670 (PMC8949360; doi:10.3390/ijerph19063670)
Supplement: Supplementary file 1 [file ijerph-19-03670-s001.zip › ijerph-1599192-supplementary.pdf]

The NeSCi Study

Neonatal unit Smoking Cessation intervention development

Draft Focus Group Topic Guide – Phase 2

Introductions

Introduce facilitators, explain:

- The purpose of the research
- Who the research is funded by
- What will happen to the information given by participants
- How the results will be disseminated

Introduce the audio recorder. Ask people to speak one at a time.

- Audio file will be destroyed after being transcribed
- No-one will be identified individually in the report

Stress confidentiality – everything said in the group is in confidence. The only reason confidentiality would be breached is if significant harm to others is mentioned. Participants should limit self-disclosure with this in mind.

Set ground rules:

- Everyone's views are valid
- It doesn't matter if people disagree
- There are no right and wrong answers
- Discussion rather than Q&A session is what we are hoping for

Ask if there are any questions

Questions for Focus Group

1. **Can you tell us about what you think might help families to keep their homes smoke-free when they take their baby home after a NICU admission?**

   Aim to establish rapport and generate lively initial discussion without prompting (prompts if necessary – support for stopping smoking? Support for relapse prevention? Information for family members about not smoking in the home?)

2. The Intervention –

Potential Component 1

Introduce the prototype intervention (show simplified overall diagram of whole intervention). Explain that this has been developed through a literature reviewing theory-based approach. Introduce first part of the intervention and how it will be delivered (e.g. Assigned nurse on NICU has one to one meeting with Mother or Father to discuss individualised needs for support, promote being smoke-free, offer cessation or relapse prevention support if required, offer signposting to ongoing support). Show diagram of how this might work.

'can you discuss now whether you think this is the right or wrong approach, and why?'

'what are your thoughts about who should be delivering the intervention?'

'when should this initial introductory part of the intervention take place?'

'should this be the same for all Mums, Dads and family members or different (tailored)?'

'can you specifically discuss NRT / e cigarettes or other options for dealing with nicotine addiction and how you think they might work for you or others?'

Potential Component 2

Introduce the second element of the prototype intervention and how it will be delivered (e.g. group information prior to leaving the NICU giving information about the benefits of smoke-free homes, advice and support)

'can you discuss now whether you think this is the right or wrong approach, and why?'

'what are your thoughts about receiving this information as part of ….?'

'when should this second part of the intervention take place?'

'how frequently should support messages be received?'

'what sorts of messages would it be helpful to receive?'

'should this be the same for all individuals, or different (tailored)?'

Potential Component 3

Introduce the third element of the prototype intervention and how it will be delivered (e.g. website with information, individualised feedback and support delivered via personalised text messaging). Show projections of screenshots to demonstrate how this might look, and examples of possible motivational or supportive text messages.

‘can you discuss now whether you think this is the right or wrong approach, and why?’

‘what are your thoughts about web based support vs. other forms of support (e.g. text messages alone , apps?)’

‘when should this third part of the intervention take place?’

‘how frequently should support messages be received?’

‘what sorts of messages would it be helpful to receive?’

‘should this be the same for all Mums, Dads and family members, or different (tailored)?’

Potential Component 4

Introduce the fourth element of the prototype intervention and how it will be delivered (e.g. Smoking cessation / relapse prevention support for partner or other family members). Discuss how this might happen – e.g. proactive contact by the stop smoking service

‘can you discuss whether you think this is the right or wrong approach, and why?’

‘what are your thoughts about whether your partner and/or any family members supporting you would engage with this approach? How would this support for their own smoking cessation be received?’

‘when should this fourth part of the intervention take place?’

‘as well as support for your partner and/or family members to quit or stay stopped from smoking, what other support do you think is needed (e.g. advice about how to support women, educational advice about the harms of SHS and THS)’

‘Should this be the same for all partners / family members, or different (tailored)?’

Potential Component 5

Introduce the fifth element of the prototype intervention and how it will be delivered (e.g. long term follow up with the Health Visitor). Discuss how this might work – e.g. Home appointment after discharge from the unit

‘can you discuss now whether you think this is the right or wrong approach, and why?’

‘do you think you personally would benefit from this type of personal follow up support?’

‘what time point would be best for a follow up visit?’

‘what are your potential concerns about this element of the intervention?’

3. ***‘is there anything else that you think should be included in the intervention?***

4. ***‘please can you discuss how long you think the intervention should continue for, and how often women should receive support?’***

5. ***‘what are the key positives and negatives of what we have discussed in your opinion?’***

6. ***‘is there anything missing from this intervention?***

7. ***‘in our next study we will need to recruit more women, and their partners to take part. Do you have any suggestions about how we might recruit women? And partners? What barriers to recruitment might there be? In theory, would you be prepared to be part of a larger randomised study? Why? Why not?’***

8. ***‘are there any other suggestions about how we might develop the intervention, or design our next study that we have not already covered?’***
